# Thyroid Function Parameters, Thyroid Status, and Vitamin B12 Deficiency in Hospitalized Adults: A Retrospective Cross-Sectional Study in Eastern India

**DOI:** 10.7759/cureus.109415

**Published:** 2026-05-22

**Authors:** Kunal Priyadarshi, Zeya Ansari, Saurabh Pathak, Nibedita Mishra, Md. Khalid Jung Khan, Sarita Kumari, Shadab Shafi, Imtiaz A Khan, Prashant Singh

**Affiliations:** 1 General Medicine, Tata Main Hospital, Jamshedpur, IND; 2 Internal Medicine, Manipal-Tata Medical College, Jamshedpur, IND; 3 Nephrology, Tata Main Hospital, Jamshedpur, IND

**Keywords:** hospitalized adults, kruskal-wallis, logistic regression, nutritional deficiency, retrospective study, subclinical hypothyroidism, thyroid function, thyroid status classification, thyroxine, vitamin b12 deficiency

## Abstract

Background: Vitamin B12 deficiency and thyroid dysfunction frequently coexist in hospitalized populations. Prior studies have not stratified patients by thyroid functional status or systematically characterized the relationship between individual thyroid parameters and B12 levels in unselected inpatients.

Aim: To evaluate associations between thyroid function parameters (triiodothyronine (T3), thyroxine (T4), thyroid-stimulating hormone (TSH)) and serum vitamin B12; compare B12 deficiency prevalence across five classified thyroid status categories; and identify independent predictors of B12 deficiency in hospitalized adults.

Methods: This retrospective cross-sectional study analyzed de-identified biochemical records from 835 unique hospitalized adults (deduplicated from 1,700 initial records) with simultaneous serum vitamin B12, T3, T4, and TSH measurements at a tertiary care hospital in eastern India. Patients were classified into five thyroid categories using established biochemical thresholds. B12 deficiency was defined as <200 pg/mL; a borderline zone (200-299 pg/mL) was additionally analyzed. Spearman's rank correlation, Mann-Whitney U test, Kruskal-Wallis test with Bonferroni correction, and multivariable binary logistic regression were performed.

Results: B12 deficiency was identified in 218 patients (26.1%). Deficiency prevalence differed significantly across thyroid categories (Chi-square p = 0.027); subclinical hypothyroid patients had the highest rate (33.5%) and overt hypothyroid patients had the lowest (16.0%). Kruskal-Wallis testing confirmed significant B12 level differences across groups (H = 27.44, p < 0.001), with overt hypothyroid patients having significantly higher median B12 than euthyroid (p_adj = 0.002) and subclinical hypothyroid (p_adj < 0.001) patients. On multivariable logistic regression, increasing age (odds ratio, OR 0.978; 95% CI 0.969-0.987; p < 0.001) and elevated T4 (OR 1.101; 95% CI 1.037-1.168; p = 0.002) were independent predictors. Pseudo R² = 0.050.

Conclusion: Vitamin B12 deficiency affects more than one in four hospitalized adults. Subclinical hypothyroidism carries the highest deficiency rate, while overt hypothyroidism paradoxically shows the lowest, the reasons for which require prospective investigation. Serum T4 and age independently predict deficiency. Prospective studies incorporating autoimmune markers and metabolic confirmatory testing are warranted.

## Introduction

Vitamin B12 (cobalamin) is an essential water-soluble micronutrient required for DNA synthesis, hematopoiesis, one-carbon metabolism, and the structural and functional integrity of the nervous system [1,2]. Deficiency produces a broad clinical spectrum - from megaloblastic anemia and peripheral neuropathy to subacute combined degeneration of the spinal cord and neuropsychiatric disturbances [1,2]. Because its clinical presentation is often insidious and diagnosis depends on a single serum biomarker, vitamin B12 deficiency remains substantially underdiagnosed, particularly among hospitalized patients [3].

Major etiologies include pernicious anemia, dietary insufficiency, malabsorption syndromes, Helicobacter pylori-associated gastric atrophy, and prolonged use of metformin or proton pump inhibitors [2,3]. The diagnostic threshold for deficiency is not universally standardized; a serum B12 <200 pg/mL is widely adopted in clinical and research settings, though a gray zone of 200-300 pg/mL is recognized, warranting confirmatory testing with methylmalonic acid (MMA) or total homocysteine (tHcy) [3,4].

Thyroid hormones exert broad metabolic effects, influencing gastrointestinal motility, erythropoiesis, and cellular micronutrient utilization [5]. Hypothyroidism impairs gastric acid secretion and gastrointestinal transit, potentially compromising the acid-dependent dissociation of protein-bound cobalamin - a prerequisite for absorption [6]. Autoimmune thyroid disease (Hashimoto thyroiditis, Graves disease) shares immunological mechanisms with pernicious anemia via autoimmune gastritis and intrinsic factor deficiency, constituting a well-characterized shared autoimmune pathway [7,8].

Several systematic reviews have documented an elevated prevalence of B12 deficiency in thyroid disease populations. Collins and Pawlak reported deficiency rates of 10%-40.5% across hypothyroid cohorts [9]. Benites-Zapata et al. reported pooled frequencies of approximately 27% in overt and subclinical hypothyroidism and 18% in autoimmune thyroid disease [10]. Individual observational studies have found even higher rates, ranging from 39.6% to 68% among autoimmune thyroid disease populations [11,12].

Despite this, a critical analytical gap persists: most prior studies were conducted in outpatient endocrine populations and categorized patients simply as hypothyroid versus euthyroid, without applying standardized biochemical cutoffs to distinguish subclinical from overt thyroid dysfunction in the same dataset. The novelty of the present study lies in its application of five-category biochemical thyroid stratification to an unselected general inpatient cohort - a population in whom thyroid and B12 tests are frequently co-ordered but rarely analyzed together with formal thyroid status classification.

The present study addresses this gap by: (1) evaluating the correlation between thyroid function parameters (T3, T4, TSH as continuous variables) and serum B12 levels; (2) classifying patients into five thyroid status categories and comparing B12 deficiency prevalence across them; (3) analyzing a clinically important borderline B12 zone (200-299 pg/mL); and (4) identifying independent predictors of B12 deficiency using multivariable logistic regression that includes both thyroid biochemical parameters and thyroid status dummies.

## Materials and methods

Study design and setting

This retrospective cross-sectional analytical study was conducted using de-identified biochemical laboratory records retrieved from the hospital laboratory information system of a tertiary care teaching hospital in eastern India, covering admissions from January 2024 to December 2025. The study was conducted in accordance with the Declaration of Helsinki. As the study utilized anonymized retrospective laboratory data without patient contact, formal ethics committee review was not required per institutional policy; institutional administrative approval was obtained prior to data access.

Serum vitamin B12 was measured by electrochemiluminescence immunoassay (ECLIA; Roche Cobas platform). Thyroid function parameters, triiodothyronine (T3), thyroxine (T4), and thyroid-stimulating hormone (TSH; labeled RTSH in the source laboratory system), were measured by the same ECLIA platform using standardized institutional reference intervals. Simultaneous B12 and thyroid function testing was performed as part of the institutional protocol for inpatients presenting with constitutional symptoms, anemia, fatigue, or unexplained neurological or metabolic disturbances, in which combined thyroid and nutritional assessment is routinely ordered.

Study population and data cleaning

An initial dataset of 1,700 laboratory-linked records from admitted adult patients who had undergone simultaneous testing for serum vitamin B12 and a complete thyroid function panel (T3, T4, TSH) was retrieved. Records were systematically deduplicated using the unique inpatient admission identifier (IP number), retaining the first record per patient per admission episode. Records with missing values in any key analyte (vitamin B12, T3, T4, or TSH) were excluded by listwise deletion. Following deduplication and exclusion of incomplete records, 835 unique patient records were included in the final analysis. Serum vitamin B12 values reported as bounded (e.g., >1,500 pg/mL) were floor-coded at their stated lower numeric bound for analysis (e.g., >1,500 coded as 1,500 pg/mL); the potential impact of this approach on correlation estimates is discussed in the Limitations section.

Inclusion and exclusion criteria

Patients were included if they were aged ≥18 years and had valid simultaneous measurements of serum vitamin B12, T3, T4, and TSH linked to a unique inpatient admission identifier. Records were excluded if the patient was aged <18 years, had missing values in any analyte, or represented duplicate admissions. Medication history, supplementation records, and dietary data were not available in the institutional laboratory dataset and were therefore not included as study variables.

Thyroid status classification

Patients were classified into five thyroid status categories using established biochemical reference thresholds consistent with international guidelines [13,14]: TSH reference range 0.4-4.0 mIU/L; T3 reference range 0.6-1.81 ng/mL; T4 reference range 4.5-12.5 µg/dL. These thresholds were applied uniformly to all patients regardless of clinical context. The thyroid status classification is presented in Table 1.

**Table 1:** Thyroid status classification Thyroid status was classified using established biochemical reference thresholds consistent with the 2016 American Thyroid Association (ATA) guidelines for hyperthyroidism [13] and the 2014 ATA guidelines for hypothyroidism [14]. Euthyroid was defined as TSH within the reference range regardless of T3 and T4. Subclinical dysfunction was defined as TSH deviation with normal T3 and T4; overt dysfunction required concurrent T3 or T4 abnormality. T3: triiodothyronine; T4: thyroxine; TSH: thyroid-stimulating hormone; mIU/L: milli-international units per liter; ng/mL: nanograms per milliliter; µg/dL: micrograms per deciliter.

Category	Biochemical Criteria
Euthyroid	TSH 0.4–4.0 mIU/L
Subclinical Hypothyroid	TSH >4.0 mIU/L, T3 and T4 within normal limits
Overt Hypothyroid	TSH >4.0 mIU/L plus T3 <0.6 ng/mL or T4 <4.5 µg/dL
Subclinical Hyperthyroid	TSH <0.4 mIU/L, T3 and T4 within normal limits
Overt Hyperthyroid	TSH <0.4 mIU/L plus T3 >1.81 ng/mL or T4 >12.5 µg/dL

Outcome variables

Vitamin B12 deficiency was defined as serum vitamin B12 <200 pg/mL, consistent with established clinical and laboratory reference thresholds [3,4]. A three-category B12 classification was additionally analyzed: deficient (<200 pg/mL), borderline (200-299 pg/mL), and normal (≥300 pg/mL).

Statistical analysis

Statistical analysis was performed using Python version 3.12 (www.python.org) with SciPy, pandas, and statsmodels libraries. Continuous variables are expressed as mean ± standard deviation (SD) or median (interquartile range, IQR) according to distribution. Normality was assessed visually using histograms. Spearman's rank correlation was used to evaluate associations between serum B12 and continuous predictors (age, T3, T4, TSH). Group comparisons between B12-deficient and non-deficient patients were conducted using the Mann-Whitney U test for continuous variables and the Chi-square test for categorical variables. Vitamin B12 levels were compared across thyroid status categories using the Kruskal-Wallis H test, followed by pairwise Mann-Whitney U tests with Bonferroni correction (10 comparisons; significance threshold p_adj < 0.05). The Chi-square test was used to compare B12 deficiency prevalence and three-category B12 status across thyroid groups.

Two multivariable binary logistic regression models were constructed with B12 deficiency (binary: <200 pg/mL vs. ≥200 pg/mL) as the outcome variable. Model 1 included age, sex, and continuous thyroid hormone parameters (T3, T4, TSH) as covariates. Model 2 additionally incorporated thyroid status categorical dummies (reference category: euthyroid), allowing assessment of whether thyroid status classification added independent predictive value beyond continuous hormone levels. McFadden Pseudo R² and likelihood ratio p-value were reported as model fit metrics. Statistical significance was set at p < 0.05. Results are presented as odds ratios (ORs) with 95% confidence intervals (CIs). A sensitivity analysis excluding floor-coded B12 values (>1,500 pg/mL, n = 253) was performed to assess the influence of ceiling-coded observations on Spearman correlation estimates.

## Results

Baseline characteristics

**Figure 1:**
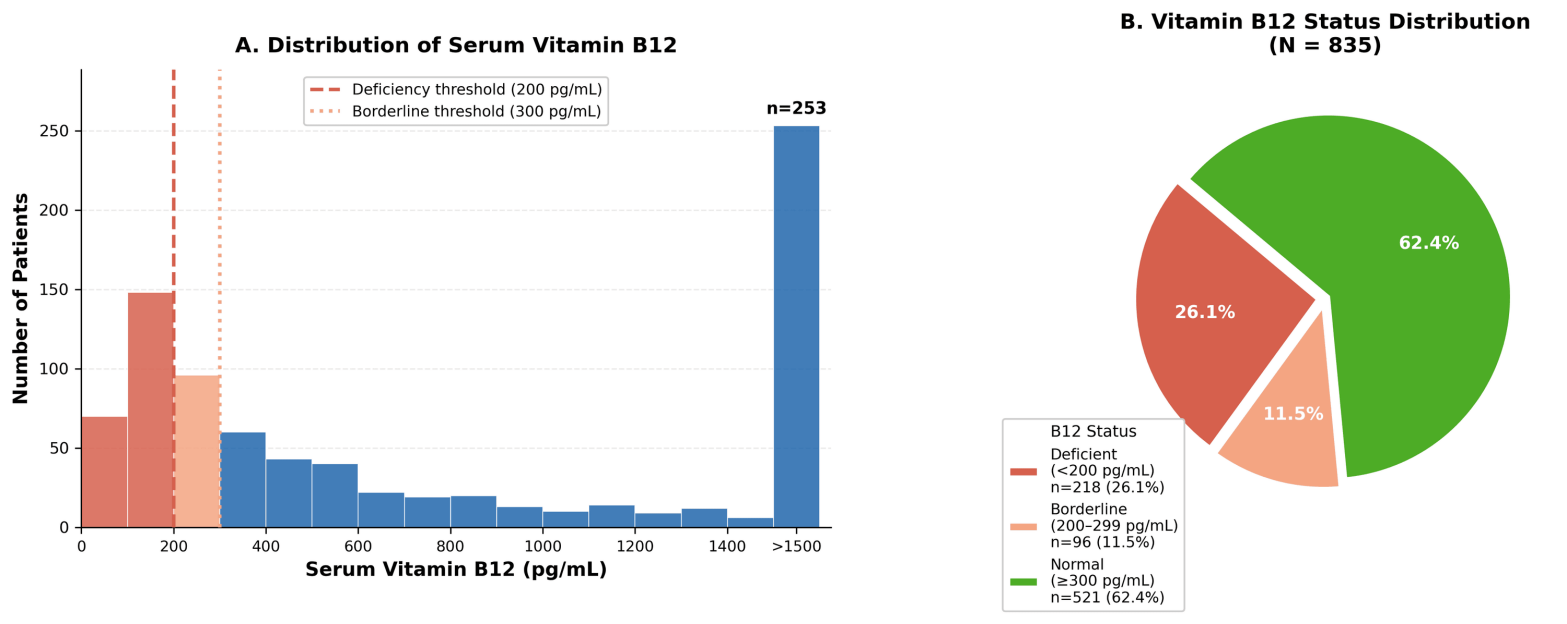
Serum vitamin B12 distribution and deficiency classification in the study population (A) Histogram of serum vitamin B12 levels (pg/mL) in 835 hospitalized adults demonstrating a right-skewed distribution. The dashed red line indicates the deficiency threshold (<200 pg/mL); the dotted orange line indicates the borderline threshold (<300 pg/mL). The bar at >1,500 pg/mL (n = 253) represents values originally reported as bounded and floor-coded at their stated lower bound for analysis. (B) Pie chart illustrating the three-category vitamin B12 classification: deficient (<200 pg/mL, n = 218, 26.1%), borderline (200–299 pg/mL, n = 96, 11.5%), and normal (≥300 pg/mL, n = 521, 62.4%). B12: vitamin B12; pg/mL: picograms per milliliter.

Of the 1,700 laboratory records initially retrieved, 835 unique patient records met the inclusion criteria after deduplication and data cleaning. The mean age was 59.69 ± 17.20 years (range: 18-102 years). Females constituted the majority at 533 (63.8%), yielding a female-to-male ratio of approximately 1.8:1. The median serum vitamin B12 level was 501 pg/mL (IQR: 194-1,500 pg/mL; mean: 739.34 ± 587.86 pg/mL), indicating a positively skewed distribution (Figure 1A). The three-category vitamin B12 classification, deficient, borderline, and normal, is shown in Figure 1B. The thyroid status distribution was: euthyroid 498 (59.6%), subclinical hypothyroid 155 (18.6%), overt hypothyroid 119 (14.3%), subclinical hyperthyroid 45 (5.4%), and overt hyperthyroid 18 (2.2%). Baseline characteristics are summarized in Table 2.

**Table 2:** Baseline demographic and biochemical characteristics (N = 835) Continuous variables expressed as mean ± standard deviation (SD) or median (interquartile range [IQR]) per distribution. Serum vitamin B12 values originally reported as bounded (e.g., >1,500 pg/mL) were floor-coded at their stated lower numeric bound for analysis. IQR: interquartile range; T3: triiodothyronine; T4: thyroxine; TSH: thyroid-stimulating hormone; pg/mL: picograms per milliliter; ng/mL: nanograms per milliliter; µg/dL: micrograms per deciliter; mIU/L: milli-international units per liter.

Variable	Value
Total patients included	835
Age (years), mean ± SD	59.69 ± 17.20
Age range (years)	18–102
Female sex, n (%)	533 (63.8%)
Male sex, n (%)	302 (36.2%)
Serum vitamin B12 (pg/mL), median (IQR)	501 (194–1,500)
Serum vitamin B12 (pg/mL), mean ± SD	739.34 ± 587.86
B12 deficient (<200 pg/mL), n (%)	218 (26.1%)
B12 borderline (200–299 pg/mL), n (%)	96 (11.5%)
B12 normal (≥300 pg/mL), n (%)	521 (62.4%)
Serum T3 (ng/mL), mean ± SD	0.73 ± 0.35
Serum T4 (µg/dL), mean ± SD	8.70 ± 3.16
Serum TSH (mIU/L), mean ± SD	6.03 ± 13.96
Euthyroid, n (%)	498 (59.6%)
Subclinical Hypothyroid, n (%)	155 (18.6%)
Overt Hypothyroid, n (%)	119 (14.3%)
Subclinical Hyperthyroid, n (%)	45 (5.4%)
Overt Hyperthyroid, n (%)	18 (2.2%)

Prevalence of vitamin B12 deficiency

**Figure 2:**
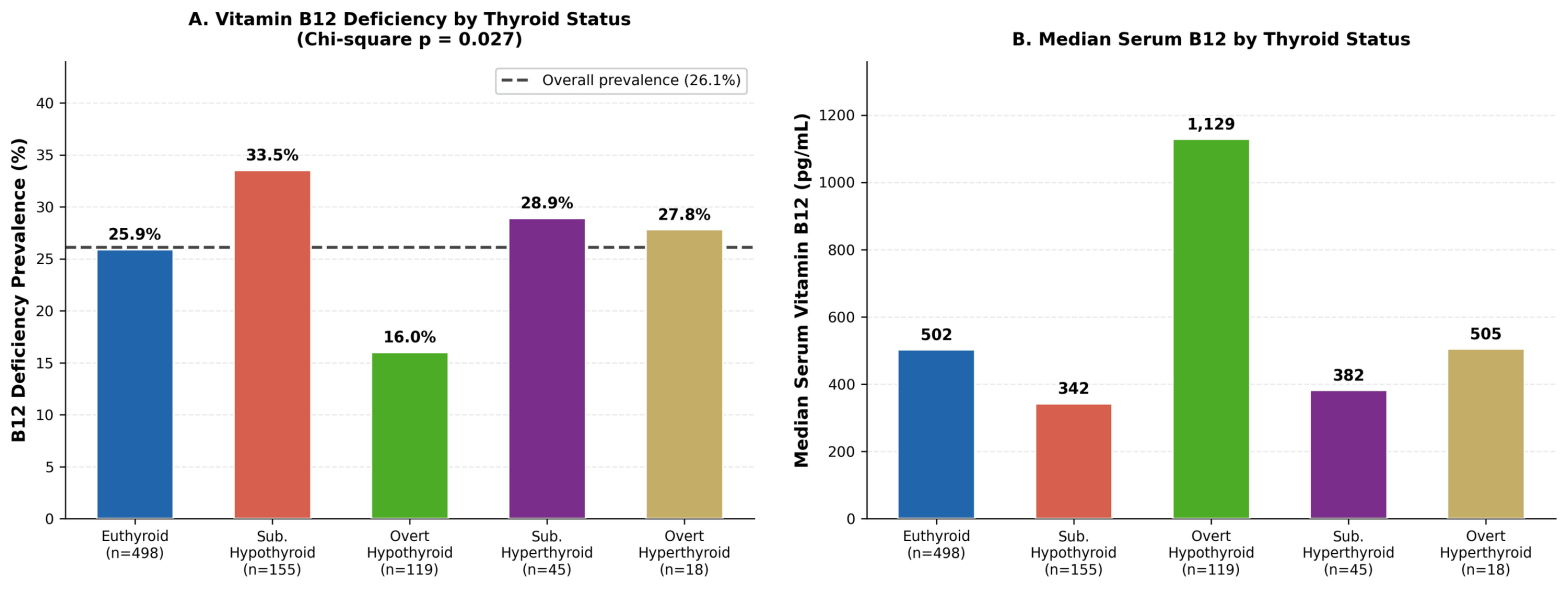
Vitamin B12 deficiency prevalence and median serum B12 levels by thyroid status category (A) Bar graph showing vitamin B12 deficiency prevalence (%) across the five thyroid status categories. The dashed gray line indicates the overall deficiency prevalence (26.1%). Subclinical hypothyroid patients (33.5%) had the highest deficiency rate; overt hypothyroid patients had the lowest (16.0%). Chi-square p = 0.027. (B) Bar graph of median serum B12 (pg/mL) by thyroid status category, illustrating the markedly elevated median B12 in the overt hypothyroid group (1,129 pg/mL) compared to all other categories. B12: vitamin B12; pg/mL: picograms per milliliter; TSH: thyroid-stimulating hormone.

Based on the threshold of <200 pg/mL, 218 patients (26.1%) were vitamin B12 deficient. An additional 96 patients (11.5%) fell in the borderline zone (200-299 pg/mL), for a combined sub-optimal B12 rate of 37.6%. Age distribution differed significantly between deficient and non-deficient groups (median age 57 vs. 64 years, Mann-Whitney p < 0.001). Sex distribution showed a non-significant trend toward a higher female proportion among deficient patients (68.8% vs. 62.1%, chi-square p = 0.090). The distribution of B12 deficiency prevalence and median serum B12 levels across the five thyroid status categories is illustrated in Figure 2.

Correlation between serum B12 and continuous variables

**Figure 3:**
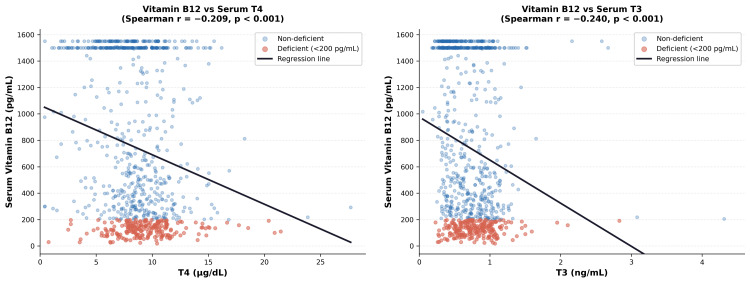
Scatter plots of serum vitamin B12 versus thyroid hormone levels Left panel: serum vitamin B12 (pg/mL) versus serum thyroxine (T4, µg/dL). Right panel: serum vitamin B12 (pg/mL) versus serum triiodothyronine (T3, ng/mL). Red points represent B12-deficient patients (<200 pg/mL); blue points represent non-deficient patients. The regression line is shown in dark blue. Weak negative correlations were observed for both T4 (Spearman r = −0.209, p < 0.001) and T3 (Spearman r = −0.240, p < 0.001). B12: vitamin B12; T3: triiodothyronine; T4: thyroxine; pg/mL: picograms per milliliter; µg/dL: micrograms per deciliter; ng/mL: nanograms per milliliter.

Spearman correlation analysis revealed a weak positive correlation between serum vitamin B12 and age (r = 0.158, p < 0.001). Weak negative correlations were identified between serum B12 and both T3 (r = −0.240, p < 0.001) and T4 (r = −0.209, p < 0.001), indicating that higher thyroid hormone concentrations were modestly associated with lower B12 levels (Figure 3). No meaningful correlation was observed between serum B12 and TSH (r = 0.011, p = 0.758). A moderate positive correlation between T3 and T4 was noted (r = 0.461, p < 0.001), indicating partial collinearity. The T3/T4 ratio did not correlate meaningfully with B12 (r = −0.056, p = 0.107). In a sensitivity analysis excluding the 253 floor-coded observations (>1,500 pg/mL), Spearman correlation coefficients for B12 versus T3 (r = −0.198) and T4 (r = −0.173) remained in the same direction and magnitude, supporting the robustness of the primary findings. Correlation coefficients are presented in Table 3.

**Table 3:** Spearman correlation matrix between serum vitamin B12 and study variables * p < 0.05. All correlation coefficients computed using Spearman's rank method. Diagonal elements (1.000) represent self-correlation. Asterisked values indicate statistically significant correlations at p < 0.05. B12: serum vitamin B12; T3: triiodothyronine; T4: thyroxine; TSH: thyroid-stimulating hormone.

Variable	B12	Age	T3	T4	TSH
Vitamin B12	1.000	0.158*	−0.240*	−0.209*	0.011
Age	0.158*	1.000	−0.283*	−0.089*	0.015
T3	−0.240*	−0.283*	1.000	0.461*	−0.070*
T4	−0.209*	−0.089*	0.461*	1.000	−0.174*
TSH	0.011	0.015	−0.070*	−0.174*	1.000

Group comparisons: B12-deficient vs. non-deficient patients

Deficient patients had significantly higher median T3 (0.76 vs. 0.66 ng/mL, p < 0.001) and T4 (9.20 vs. 8.41 µg/dL, p < 0.001), and lower median age (57 vs. 64 years, p < 0.001) compared to non-deficient patients. Median TSH did not differ significantly between groups (2.62 vs. 2.60 mIU/L, p = 0.802). Sex distribution showed a non-significant trend toward a higher female proportion among deficient patients (68.8% vs. 62.1%, p = 0.090). These findings are detailed in Table 4.

**Table 4:** Group comparison: B12-deficient vs. non-deficient patients Mann-Whitney U test used for continuous variables; Chi-square test used for sex. All p-values are two-sided. IQR: interquartile range; T3: triiodothyronine; T4: thyroxine; TSH: thyroid-stimulating hormone; pg/mL: picograms per milliliter; ng/mL: nanograms per milliliter; µg/dL: micrograms per deciliter; mIU/L: milli-international units per liter.

Variable	B12 Deficient (n = 218)	Non-Deficient (n = 617)	p-value
Age (years), median (IQR)	57 (39–69)	64 (53–73)	<0.001
T3 (ng/mL), median (IQR)	0.76 (0.58–0.98)	0.66 (0.44–0.90)	<0.001
T4 (µg/dL), median (IQR)	9.20 (7.86–10.80)	8.41 (6.60–10.31)	<0.001
TSH (mIU/L), median (IQR)	2.62 (1.27–5.28)	2.60 (1.27–5.33)	0.802
Female sex, n (%)	150 (68.8%)	383 (62.1%)	0.090

Vitamin B12 deficiency across thyroid status categories

The prevalence of vitamin B12 deficiency differed significantly across thyroid status categories (Chi-square p = 0.027). Subclinical hypothyroid patients had the highest deficiency rate (33.5%), followed by subclinical hyperthyroid (28.9%), overt hyperthyroid (27.8%), and euthyroid (25.9%); overt hypothyroid patients had the lowest rate (16.0%). When the three-category B12 classification (deficient / borderline / normal) was applied, the distribution across thyroid groups also reached statistical significance (Chi-square p = 0.028).

**Figure 4:**
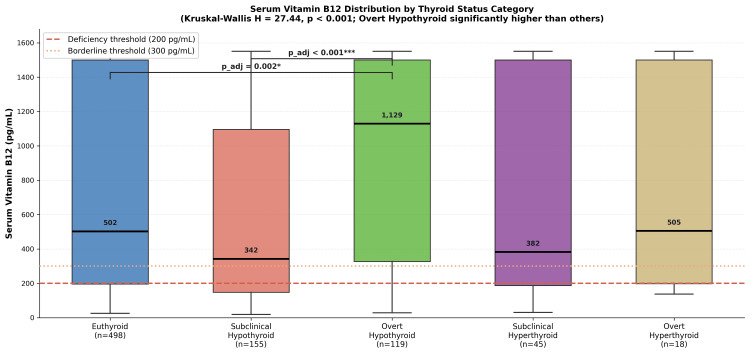
Box plots of serum vitamin B12 levels by thyroid status category Box plots display the median, interquartile range, and distribution of serum vitamin B12 (pg/mL) across the five thyroid status categories (N = 835). Median values annotated above each box: euthyroid 502, subclinical hypothyroid 342, overt hypothyroid 1,129, subclinical hyperthyroid 382, overt hyperthyroid 505 pg/mL. The dashed horizontal line at 200 pg/mL indicates the deficiency threshold; the dotted horizontal line at 300 pg/mL indicates the borderline threshold. Overt hypothyroid patients had significantly higher median serum B12 than euthyroid (p_adj = 0.002) and subclinical hypothyroid (p_adj < 0.001) patients on Bonferroni-corrected pairwise comparisons. Kruskal-Wallis H = 27.44, p < 0.001. B12: vitamin B12; pg/mL: picograms per milliliter; IQR: interquartile range; p_adj: Bonferroni-corrected p-value.

Kruskal-Wallis testing confirmed significant differences in serum B12 levels across thyroid status groups (H = 27.44, p < 0.001; Figures 2, 4). Bonferroni-corrected pairwise comparisons revealed that overt hypothyroid patients had significantly higher median serum B12 than both euthyroid patients (p_adj = 0.002) and subclinical hypothyroid patients (p_adj < 0.001). No other pairwise comparisons reached corrected significance. Findings in the subclinical hyperthyroid (n = 45) and overt hyperthyroid (n = 18) subgroups should be considered exploratory given the small group sizes. These findings are summarized in Table 5.

**Table 5:** Vitamin B12 status by thyroid category (N = 835) Chi-square test applied to compare B12 deficiency prevalence (binary: deficient vs. non-deficient) across the five thyroid status categories (chi-square p = 0.027). Three-category B12 distribution also significantly differed across groups (chi-square p = 0.028). Kruskal-Wallis H test used to compare continuous serum B12 levels across groups (H = 27.44, p < 0.001). Bonferroni-corrected pairwise comparisons (10 comparisons; significance threshold p_adj < 0.05): Overt Hypothyroid vs. Euthyroid p_adj = 0.002*; Overt Hypothyroid vs. Subclinical Hypothyroid p_adj < 0.001*; all other pairwise comparisons non-significant after correction. IQR: interquartile range; pg/mL: picograms per milliliter.

Thyroid Status	N	Deficient n (%)	Borderline n (%)	Normal n (%)	Median B12 (IQR), pg/mL
Euthyroid	498	129 (25.9%)	62 (12.4%)	307 (61.6%)	502 (196–1,500)
Subclinical Hypothyroid	155	52 (33.5%)	18 (11.6%)	85 (54.8%)	342 (148–1,096)
Overt Hypothyroid	119	19 (16.0%)	8 (6.7%)	92 (77.3%)	1,129 (328–1,500)
Subclinical Hyperthyroid	45	13 (28.9%)	5 (11.1%)	27 (60.0%)	382 (187–1,500)
Overt Hyperthyroid	18	5 (27.8%)	3 (16.7%)	10 (55.6%)	505 (198–1,500)
Chi-square p-value	—	0.027	—	—	Kruskal-Wallis p < 0.001

Multivariable logistic regression analysis

Two multivariable logistic regression models were constructed to identify independent predictors of vitamin B12 deficiency. In Model 1 (continuous thyroid parameters only; N = 835), each additional year of age was associated with a 2.2% reduction in the odds of B12 deficiency (OR 0.978; 95% CI 0.969-0.987; p < 0.001), indicating that deficiency was paradoxically less likely with increasing age in this hospitalized cohort, a finding discussed further in the Discussion section. Elevated serum T4 was independently associated with increased odds of deficiency (OR 1.101; 95% CI 1.037-1.168; p = 0.002). Serum T3 (OR 1.164; 95% CI 0.702-1.930; p = 0.555) and male sex (OR 0.915; 95% CI 0.648-1.292; p = 0.615) did not reach statistical significance. TSH reached borderline significance (OR 1.012; 95% CI 1.002-1.023; p = 0.024). Model 1 fit: Pseudo R² = 0.050, LR p < 0.001.

**Figure 5:**
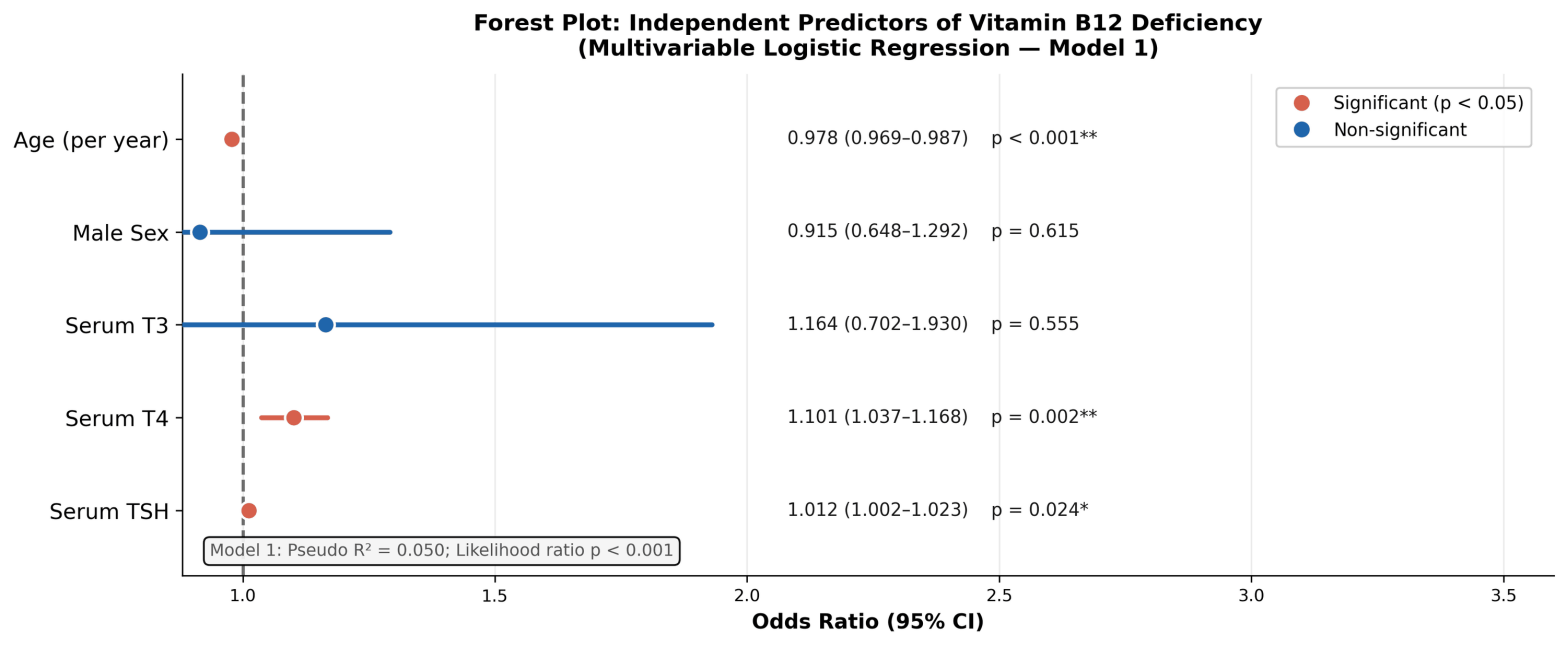
Forest plot of odds ratios from multivariable logistic regression for vitamin B12 deficiency (Model 1) Odds ratios (95% confidence intervals) for each predictor in Model 1, which included age, sex, and continuous thyroid hormone parameters (T3, T4, TSH). Statistically significant predictors (p < 0.05) were increasing age (OR 0.978; 95% CI 0.969–0.987; p < 0.001), elevated serum T4 (OR 1.101; 95% CI 1.037–1.168; p = 0.002), and serum TSH (OR 1.012; 95% CI 1.002–1.023; p = 0.024). Serum T3 and male sex did not reach statistical significance. The dashed vertical line at OR = 1.0 represents the null effect. Model fit: Pseudo R² = 0.050; likelihood ratio p < 0.001. OR: odds ratio; CI: confidence interval; T3: triiodothyronine; T4: thyroxine; TSH: thyroid-stimulating hormone; B12: vitamin B12.

In Model 2 (continuous parameters plus thyroid status dummies; N = 835), the significance of age (OR 0.979; 95% CI 0.970-0.988; p < 0.001) and T4 (OR 1.107; 95% CI 1.038-1.181; p = 0.002) was preserved. TSH reached full significance in Model 2 (OR 1.017; 95% CI 1.004-1.029; p = 0.009). None of the thyroid status dummies (subclinical hypothyroid, overt hypothyroid, subclinical hyperthyroid, overt hyperthyroid, each vs. euthyroid) reached statistical significance after adjustment for continuous hormone levels. Model 2 fit: Pseudo R² = 0.058, LR p < 0.001. These results are presented in Table 6 and Figure 5.

**Table 6:** Multivariable logistic regression: independent predictors of vitamin B12 deficiency * p < 0.05; ** p < 0.01. Model 1 included age, sex, and continuous thyroid hormone parameters (T3, T4, TSH) as predictors. Model 2 additionally incorporated thyroid status categorical dummies (reference category: euthyroid). Reference category for sex: female. McFadden Pseudo R² reported as model fit metric. OR: odds ratio; CI: confidence interval; T3: triiodothyronine; T4: thyroxine; TSH: thyroid-stimulating hormone; LR: likelihood ratio.

Predictor	Model 1 OR (95% CI)	p	Model 2 OR (95% CI)	p
Age (per year)	0.978 (0.969–0.987)	<0.001**	0.979 (0.970–0.988)	<0.001**
Male sex	0.915 (0.648–1.292)	0.615	0.901 (0.637–1.274)	0.555
Serum T3	1.164 (0.702–1.930)	0.555	1.131 (0.663–1.929)	0.652
Serum T4	1.101 (1.037–1.168)	0.002**	1.107 (1.038–1.181)	0.002**
Serum TSH	1.012 (1.002–1.023)	0.024*	1.017 (1.004–1.029)	0.009**
Subclinical Hypothyroid (vs. Euthyroid)	—	—	1.153 (0.756–1.757)	0.508
Overt Hypothyroid (vs. Euthyroid)	—	—	0.570 (0.300–1.086)	0.088
Subclinical Hyperthyroid (vs. Euthyroid)	—	—	1.253 (0.630–2.491)	0.520
Overt Hyperthyroid (vs. Euthyroid)	—	—	0.469 (0.138–1.601)	0.227
Pseudo R²	0.050	LR p < 0.001	0.058	LR p < 0.001

## Discussion

The present retrospective cross-sectional study evaluated the relationship between thyroid function parameters, thyroid status classification, and vitamin B12 deficiency in 835 unique hospitalized adults. Four principal findings emerge: (1) vitamin B12 deficiency was prevalent in more than one in four patients, with combined sub-optimal B12 (deficient + borderline) affecting more than one in three; (2) vitamin B12 deficiency prevalence and serum B12 levels differed significantly across thyroid status categories; (3) serum T4 and age were independent predictors of deficiency on multivariable analysis; and (4) the low Pseudo R² (5.0%-5.8%) across both models confirms that thyroid parameters have limited standalone utility for predicting B12 deficiency, underscoring the necessity of direct biochemical screening. All mechanistic explanations offered in this Discussion should be considered hypothesis-generating, as key confounders, including medication use, supplementation history, and dietary intake, were not available in this dataset.

Prevalence of vitamin B12 deficiency

The overall deficiency prevalence of 26.1% is consistent with prior estimates across institutional and endocrine populations. Collins and Pawlak reported rates of 10%-40.5% in hypothyroid cohorts [9]. Benites-Zapata et al. reported pooled frequencies of approximately 27% across overt and subclinical hypothyroid groups [10]. Our finding of 37.6% combined sub-optimal B12 (deficient + borderline) is clinically relevant, as borderline B12 values are associated with elevated MMA and tHcy even in the absence of overt deficiency [15,16]. Prospective studies should incorporate metabolic confirmatory testing in borderline cases to define the true prevalence of functional B12 deficiency in this population.

The subclinical dysfunction paradox

A noteworthy observation is that subclinical hypothyroid patients (33.5%) had higher B12 deficiency rates than overt hypothyroid patients (16.0%). This pattern is counterintuitive and was not hypothesized a priori. We offer two possible, but unmeasured and therefore unconfirmed, explanations for this finding. First, patients with established overt hypothyroidism are more likely to have received a prior thyroid diagnosis, potentially prompting concurrent nutritional evaluation and B12 supplementation before or at admission. Second, patients with subclinical dysfunction may not yet have been diagnosed or treated, leaving coexisting nutritional deficiencies unaddressed. However, as treatment status, supplementation records, and medication history were unavailable in this dataset, these explanations are purely speculative and cannot be tested with the current data. Whether the overt hypothyroid group had received prior B12 supplementation or levothyroxine therapy that indirectly influenced B12 status remains the primary unanswered question and warrants prospective investigation with detailed medication and supplementation records.

The subclinical hyperthyroid finding (28.9% deficiency rate) is also noted. One biologically plausible, but unmeasured, mechanism is that elevated metabolic activity associated with excess thyroid hormone may accelerate cobalamin utilization. However, this remains a hypothesis without supporting data in this study, and the small subgroup size (n = 45) limits the reliability of this estimate [17].

Serum T4 as an independent predictor

Elevated serum T4 was independently associated with increased odds of B12 deficiency in both regression models (Model 1 OR 1.101; Model 2 OR 1.107 per unit increase; both p ≤ 0.002). This finding is consistent with prior observations by Al-Khamis and Al-Haddad in endocrine populations [18]. The mechanism underlying this association cannot be determined from this dataset. Elevated T4 may reflect greater thyroid hormone bioactivity or may serve as a nonspecific marker of physiologic stress in acutely ill inpatients in whom nutritional compromise is more common [19]. The persistence of T4 significance in Model 2 after adjustment for thyroid status classification suggests that continuous T4 captures clinically relevant variation beyond that captured by categorical classification alone.

TSH demonstrated significance in Model 2 (OR 1.017; p = 0.009) and borderline significance in Model 1 (OR 1.012; p = 0.024), contrasting with the negligible bivariate correlation (r = 0.011). This discordance may reflect non-linearity or confounding by thyroid status that is not fully captured by the dummy variables. Importantly, in hospitalized patients, TSH values may be altered by non-thyroidal illness syndrome (NTIS), also known as euthyroid sick syndrome, in which acute systemic illness suppresses TSH and alters T3 and T4 levels independent of intrinsic thyroid disease [20]. The contribution of NTIS to the observed TSH associations cannot be excluded in this study and represents a significant source of potential misclassification. These findings are consistent with prior observations by Al-Mousawi et al. of context-dependent TSH-B12 relationships in thyroid populations [21].

The paradoxical relationship between age and B12 deficiency

Each additional year of age was associated with a 2.2% reduction in the odds of B12 deficiency (OR 0.978 per year; p < 0.001), indicating that B12 deficiency was paradoxically less common among older inpatients in this cohort. This is counterintuitive relative to the general population literature, in which older age is established as a risk factor for B12 deficiency due to gastric atrophy, impaired intrinsic factor secretion, and age-related malabsorption [22,23]. This apparent reversal is most plausibly explained by features of the inpatient setting: older hospitalized patients in this institution may be more frequently prescribed nutritional supplementation as part of routine inpatient care, and are more likely to have had prior B12 deficiency identified and corrected before the index admission. Additionally, referral bias may enrich younger inpatients with clinically suspected deficiency-related presentations such as peripheral neuropathy, megaloblastic anemia, or neuropsychiatric symptoms, conditions that prompt targeted B12 testing. This age paradox has been observed in at least one prior inpatient study [12]. These explanations, however, remain unverified in the absence of supplementation and medication data.

Implications of thyroid status classification

The significant variation in B12 deficiency prevalence across thyroid status categories, confirmed by both Chi-square and Kruskal-Wallis testing, supports the value of five-category biochemical thyroid stratification when studying the thyroid-B12 relationship, rather than relying solely on continuous hormone values or a binary hypothyroid versus euthyroid classification. The absence of significant thyroid status dummy coefficients in Model 2 after adjustment for continuous T3, T4, and TSH suggests that continuous hormone values mediate the categorical effect. Clinically, this means that both continuous thyroid parameters and categorical thyroid status provide complementary information, and neither should be used in isolation.

Limitations

Several limitations warrant acknowledgment. First, and most critically, medication data, including levothyroxine, metformin, proton pump inhibitors, and vitamin B12 supplementation, were unavailable from the institutional laboratory dataset. Metformin and proton pump inhibitors are well-established causes of B12 depletion; levothyroxine therapy may independently influence gastrointestinal function and thereby B12 absorption. The absence of these data is the primary constraint on all mechanistic interpretations in this study, which must be considered hypothesis-generating rather than explanatory. Second, the retrospective cross-sectional design precludes causal inference. Third, single-center origin limits external generalizability. Fourth, dietary intake, gastrointestinal disease status, and autoimmune antibody profiles (anti-thyroid peroxidases (TPO), anti-intrinsic factor) were unavailable, precluding adjustment for key confounders of both thyroid and B12 status. Fifth, B12 deficiency was defined solely by serum B12 <200 pg/mL without confirmatory metabolic biomarkers (methylmalonic acid (MMA), total homocysteine (tHcy)), which may misclassify patients in the borderline zone. Sixth, bounded B12 values (>1,500 pg/mL, n = 253, 30.3%) were floor-coded, which underestimates true B12 levels in the upper tail; sensitivity analysis excluding these values confirmed that Spearman correlations remained directionally consistent, though attenuated in magnitude. Seventh, thyroid status classification was based on biochemical parameters without clinical diagnosis data, precluding separation of treated from untreated thyroid disease. Eighth, this study was conducted in a general inpatient population in whom non-thyroidal illness syndrome (NTIS) may suppress TSH and alter T3 and T4 values independent of intrinsic thyroid disease, potentially introducing thyroid status misclassification and confounding TSH-B12 associations. Ninth, total T3 and T4 measurements were used as available from the institutional laboratory system. Total hormone levels may poorly reflect thyroid hormone bioavailability in hospitalized patients due to alterations in thyroid-binding proteins secondary to acute illness; free T3 and free T4 measurements would be more appropriate in future prospective studies. Tenth, the subclinical hyperthyroid (n = 45) and overt hyperthyroid (n = 18) subgroups were small; estimates in these categories should be considered exploratory and interpreted with caution. Eleventh, the moderate T3-T4 collinearity (r = 0.461) may affect regression coefficient stability.

## Conclusions

Vitamin B12 deficiency is prevalent in more than one in four hospitalized adults, with combined sub-optimal B12 status affecting more than one in three. Thyroid status classification reveals a clinically important pattern: subclinical hypothyroidism is associated with the highest B12 deficiency prevalence (33.5%), while overt hypothyroidism shows the lowest rate (16.0%). The reasons for this pattern are not determinable from the current dataset and require prospective investigation with medication, supplementation, and dietary data. Serum T4 and age independently predict B12 deficiency on multivariable analysis; however, the overall explanatory power is modest (Pseudo R² = 0.050), and thyroid function tests should not be used as surrogate markers for B12 deficiency in routine clinical practice.

Clinicians should rely on direct biochemical screening guided by individual clinical risk factors - including dietary history, gastrointestinal disease, medication use (metformin, proton pump inhibitors), and autoimmune disease status. Prospective multicentric studies incorporating free thyroid hormone measurements, autoimmune antibody profiles (anti-TPO, anti-intrinsic factor), supplementation records, metabolic confirmatory tests (MMA, homocysteine), NTIS assessment, and dietary data are warranted to elucidate the causal and mechanistic relationships between thyroid functional status and vitamin B12 metabolism.
